# Proteogenomic analysis of pancreatic cancer subtypes

**DOI:** 10.1371/journal.pone.0257084

**Published:** 2021-09-10

**Authors:** Doris Kafita, Panji Nkhoma, Mildred Zulu, Musalula Sinkala

**Affiliations:** 1 Department of Biomedical Sciences, School of Health Sciences, University of Zambia, Lusaka, Zambia; 2 Department of Pathology and Microbiology, School of Medicine, University of Zambia, Lusaka, Zambia; Centro Nacional de Investigaciones Oncologicas, SPAIN

## Abstract

Pancreatic cancer remains a significant public health problem with an ever-rising incidence of disease. Cancers of the pancreas are characterised by various molecular aberrations, including changes in the proteomics and genomics landscape of the tumour cells. Therefore, there is a need to identify the proteomic landscape of pancreatic cancer and the specific genomic and molecular alterations associated with disease subtypes. Here, we carry out an integrative bioinformatics analysis of The Cancer Genome Atlas dataset, including proteomics and whole-exome sequencing data collected from pancreatic cancer patients. We apply unsupervised clustering on the proteomics dataset to reveal the two distinct subtypes of pancreatic cancer. Using functional and pathway analysis based on the proteomics data, we demonstrate the different molecular processes and signalling aberrations of the pancreatic cancer subtypes. In addition, we explore the clinical characteristics of these subtypes to show differences in disease outcome. Using datasets of mutations and copy number alterations, we show that various signalling pathways previously associated with pancreatic cancer are altered among both subtypes of pancreatic tumours, including the Wnt pathway, Notch pathway and PI3K-mTOR pathways. Altogether, we reveal the proteogenomic landscape of pancreatic cancer subtypes and the altered molecular processes that can be leveraged to devise more effective treatments.

## Introduction

Pancreatic cancer remains one of the deadliest malignancies [1,2]. Its incidence has continued to increase over the last few years because of lifestyle shifts in population and increased life expectancy [3–6]. The molecular characterization of pancreatic cancer, including the transcriptomic, genomic, and epigenetic landscape, has been studied by large-scale molecular profiling projects and many other studies [7–14]. Gene expression changes have led to the identification of molecular subtypes of the disease, which have treatment and prognostic importance [15–20]. Many mutations and copy number changes are now known to drive pancreatic oncogenesis, and disease aggressiveness and alteration in various signal transduction pathways have now been identified [21–24].

Studies of many cancers, including those of the breast, ovary and colon, have shown that the transcriptome is poorly correlated to the proteome, with only 10%-20% of the variation in protein level explained by mRNA transcription levels [25,26]. Recent studies have conducted a proteomic analysis of pancreatic cancer [27–33]; however, they have not linked the proteomic subtypes with alterations in various signalling pathways to the specific cancer gene mutations and/or the disease outcomes of the afflicted patients.

## Results

### Proteomics subtypes of pancreatic cancer

**Fig 1:**
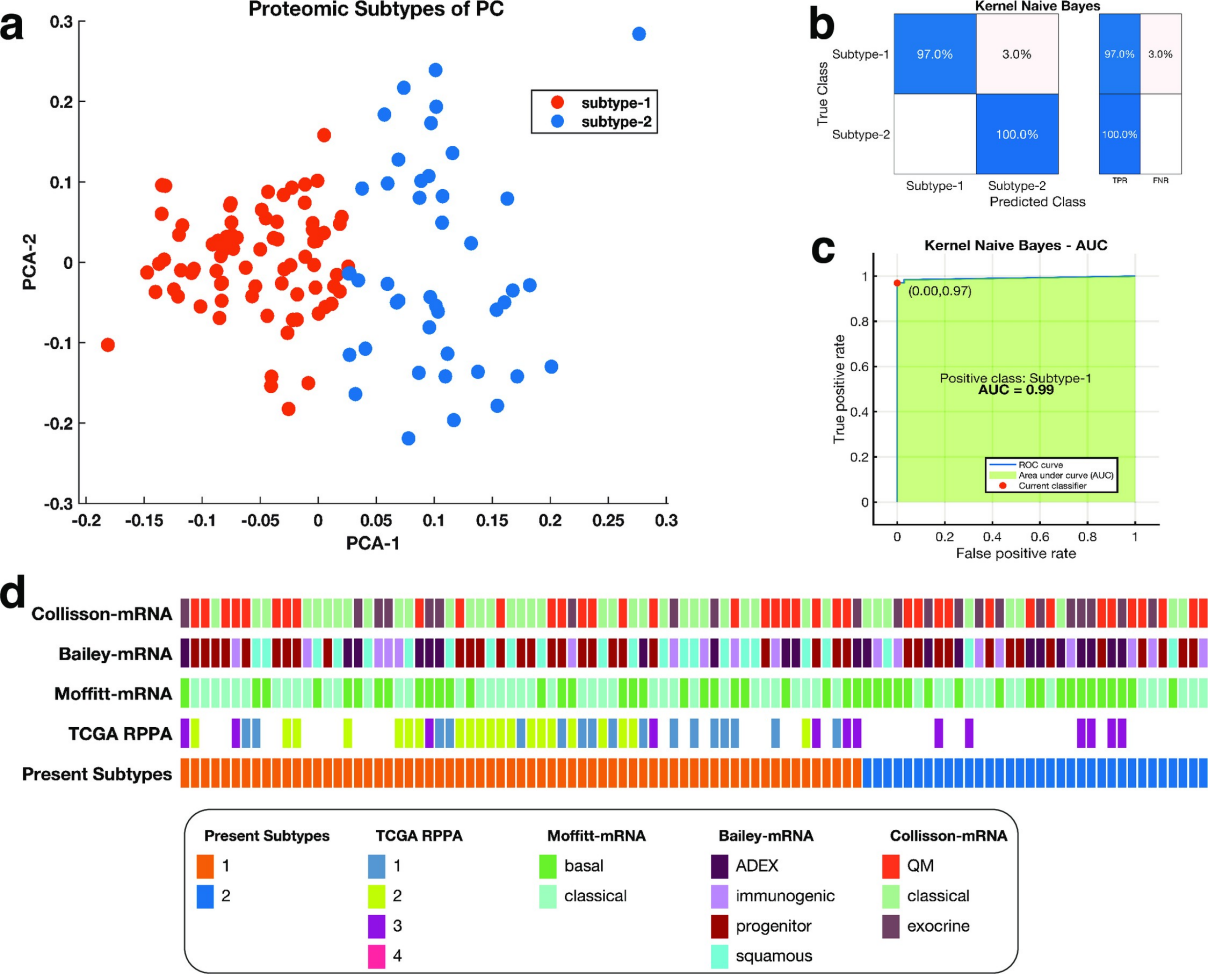
**(a)** Clustering of pancreatic tumours; the first and second principal components of a PCA analysis are plots on the x-axis and y-axis response. The points are coloured according to the K-mean clustering defined cluster assignments. **(b)** a representative confusion matrix for the Kernel naïve Bayes classifier used to validate the clustering of the proteomic subtypes of pancreatic cancer. The blue cells correspond to samples that are correctly classified. The red cells correspond to incorrectly classified samples. In the plot, TFR shows the true-positive rate and TNR indicate the false-negative rate. **(c)** the Receiver operating characteristic Curve for the Kernel naïve Bayes. The green shaded area represents the area under the curve (AUC = 0.99). **(d)** Comparison between the current proteomic based classification of pancreatic cancers to other classification schemes from top to bottom: mRNA-based classification schemes established by Collisson et al.; Bailey et al.; and Moffitt et al., and the TCGA’s [23] RPPA classification scheme.

We applied unsupervised K-means clustering with a squared Euclidean metric to the proteomics data of the pancreatic cancer samples available in the TCGA to identify two consistent clusters of patients tumours (Fig 1A) [34]. One cluster comprised 67 tumour samples that we named as subtype-1, and the other cluster comprised 34 tumour samples which we named subtype-2. Furthermore, we evaluated the reproducibility of our two-cluster classification of pancreatic tumours using a supervised machine learning classification approach. Here, we used a Kernel naïve Bayes method [35] to show that we could accurately predict tumour subtypes with an accuracy of 98% (Fig 1B) and area under the curve of 0.97 (Fig 1C). Furthermore, we compared the current proteomics subtypes of pancreatic cancer to the expression subtypes previously described in the literature [23]. Here, we found that the subtypes-1 and subtype-2 tumours were not consistently classified into any previously described expression subtypes of pancreatic cancer (Fig 1D).

### Clinical characteristics of the proteomic subtypes of pancreatic cancer

**Fig 2:**
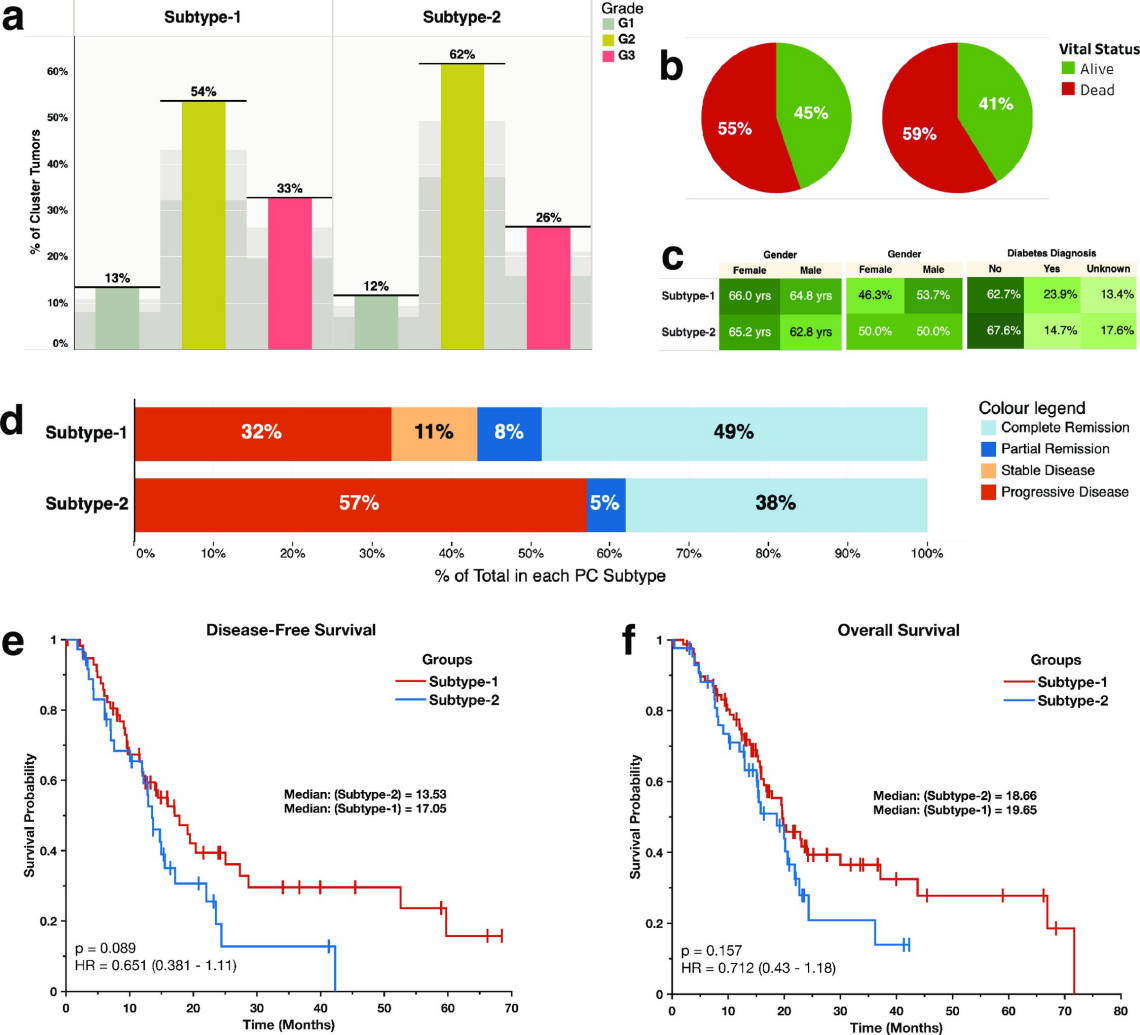
**(a)** Distribution of tumour grades across the proteomic subtypes: Showing the percentage of the total count of the number of tumours for each grade of tumour broken down by proteomic subtype. **(b)** Pie chart showing the vital statistics after the first course of treatment across the two disease subtypes. **(c)** Highlight tables showing the distribution of, from left to right: The study participants’ age, gender, and the diabetes diagnosis. **(d)** The clinical outcomes after the first course of treatment across the disease subtypes. **(e)** Kaplan-Meier curve of the disease-free survival months of patients afflicted by each pancreatic cancer subtype (**f)** Kaplan-Meier curve of the overall survival months of patients afflicted by each pancreatic cancer subtype.

We found a similar distribution of tumours of various grades (Fig 2A) and the patients living at the end of follow up (Fig 2B) for each of the two pancreatic cancer subtypes. Furthermore, we found similar distributions in the age, gender, and the diagnosis of diabetes between the patients afflicted with the two subtypes of pancreatic cancer (Fig 2C).

We found that of the patients afflicted with subtype-1 tumours, 38% were disease-free at the end of the follow-up, whereas 62% of the patients had a disease that either progressed or recurred, with only 19% of these patients surviving by the end of the follow-up period. For patients afflicted with subtype-2 tumours, 31% were disease-free at the end of the follow-up, whereas 57% of the patients had a disease that either progressed or recurred, with only 17% of them surviving by the end of the follow-up periods (Fig 2D).

However, after comparing the median disease-free survival (DFS) period, we found that the duration was shorter for patients afflicted with subtype-2 tumours (DFS = 13.5 months) than for patients with subtype-1 tumours (DFS = 17.1 months; Fig 1E). However, we found no statistically significant difference (Kaplan-Meier test; p = 0.089) in the duration of the DFS between the pancreatic cancer subtypes. Likewise, the overall survival (OS) periods for the patients with subtype-2 tumours (OS = 18.7 months) were not significantly different (Kaplan-Meier test; p = 0.157) to those of subtype-1 tumours (OS = 19.7 months; Fig 1F) [36].

### Altered signalling pathways and molecular processes distinguish disease subtypes

Next, we compared the expression levels of various proteins between the two disease subtypes. We found that the subtype-1 tumours expressed significantly higher levels of several proteins, including mTOR, E-Cadherin, and Raf-pS338, compared to the subtype-2 tumours (S1 File). Conversely, the subtype-2 tumours expressed significantly higher levels of several proteins, including Stathmin, Mre11 and MAP2K1, than the subtype-1 tumours (S1 File).

**Fig 3:**
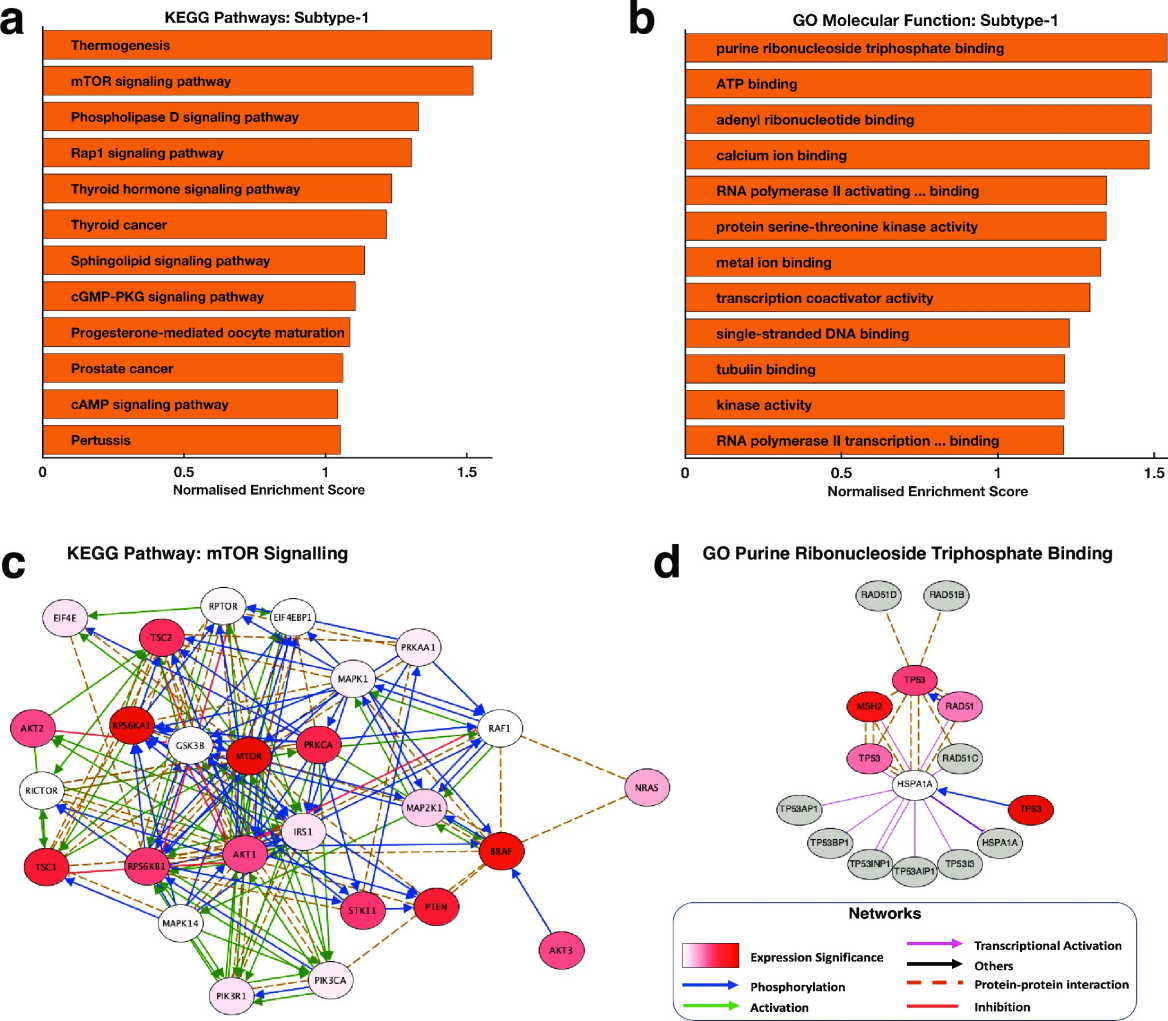
Showing the top-ranked enriched **(a)** KEGG pathway and **(b)** GO Molecular functions in the subtype-1 tumours compared to the subtype-2 tumours. **(c)** A network of the genes that encompass the KEGG pathways “mTOR signalling pathway” that we found significantly enriched in subtype-1 tumours compared to subtype-2 tumours. The nodes are coloured using the degree of statistical significance for each protein (negative logarithm of the p-values) between subtype-1 and subtype-2 tumours. **(d)** A network of the genes that encompass the GO-term molecular function “Purine Ribonucleoside Triphosphate Binding” that we found significantly enriched in the subtype-1 compared to the subtype-2 tumours. The nodes are coloured based on the degree of statistical significance for each protein (negative logarithm of the p-values) between subtype-1 and subtype-2 tumours with redder colours indicating a higher level of statistical significance.

We applied Gene Set Enrichment Analysis (GSEA) [37] to extract knowledge of the KEGG pathways and Gene Ontology (GO) Molecular Function terms that are enriched for in the subtypes-1 tumours compared to the subtype-2 tumours. We found that the two KEGG pathways most significantly enriched for in subtype-1 tumours were those involving thermogenesis and mTOR signalling (Fig 3A). Furthermore, within the mTOR signalling pathway, we found several oncogenes, including mTOR, AKT1 and AKT2 and tumour suppressor genes that were significantly upregulated for subtype-1 tumours and all of which have previously been linked to pancreatic carcinogenesis (S1 and S2 Files) [38–40].

Among the GO molecular processes, we found that the subtype-1 tumours were enriched for, among others, the GO terms associated Purine Nucleotide Triphosphate Binding, ATP Binding and among others (Fig 3B, also see S2 File).

Overall, our findings revealed the distinct molecular mechanisms by which the development and progression of subtype-1 and subtype-2 may occur. For example, the KEGG pathway “*mTOR signalling*” forms a network whose nodes are significantly upregulated in subtype-1 tumours and are previously linked to oncogenesis, but in this case, we suggest that this is likely only correct in subtype-1 tumours and not in subtype-2 tumours (Fig 3C). We also identified proteins involved in the GO term “*Purine Ribonucleoside Triphosphate Binding*”, a molecular process that may only play an essential role in the oncogenesis of the subtype-1 tumours (Fig 3D).

### The mutational landscape of proteomics subtypes of pancreatic cancer

**Fig 4:**
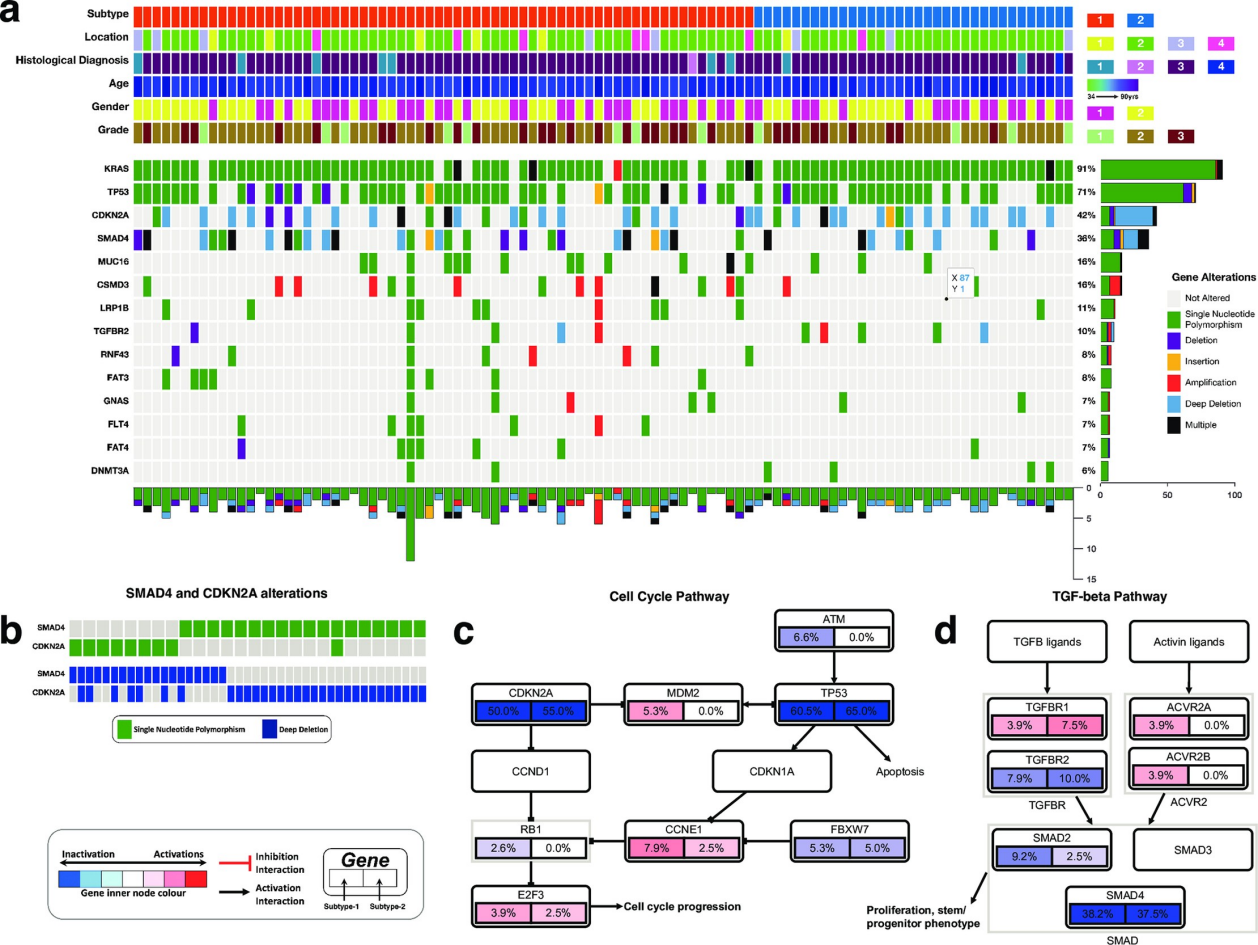
**(a)** The integrated plot of gene mutations, copy number alterations and the clinical features of the pancreatic tumours and the afflicted patients. From top to bottom panels indicate the proteomic subtypes of pancreatic cancer; the tumour location in the pancreas; the histological subtypes of the tumours; age at diagnosis; the patient’s gender; the tumour’s histological grade; non-silent mutations and copy number alteration frequency in each tumour across the altered genes. The key to the number coding of tumour location is 1; head, 2; body, 3; other, 4; tail. The number coding of histological diagnosis is 1; Pancreas-Adenocarcinoma-Other Subtype, 2; Pancreas-Colloid (mucinous non-cystic) Carcinoma, 3; Pancreatic Ductal Adenocarcinoma, 4; Discrepancy. **(b)** Mutual exclusivity of SMAD4 and CDKN2A mutations. **(c)** Gene alterations in the cell cycle pathways genes. **(d)** Gene alterations in the TGF-beta pathway genes.

We evaluated the extent of gene mutations and copy number variations (which we collectively refer to as gene alterations) in pancreatic cancer. Focusing only on the Consensus Cancer Genes [41], we found no significant difference in the gene alteration spectrum between the two pancreatic cancer subtypes (Fig 4A and S2 File). Across the disease subtypes, we revealed that, as previously reported by others, the most frequently altered genes were KRAS (altered in 91% of all samples), TP53 (71%), CDKN2A (42%) and SMAD4 (36%); Fig 4A [2,16,42].

Interestingly, we observed that the mutations in SMAD4 and those in CDKN2A tended towards being mutually exclusive (co-occurrence odd ratio = -0.137, p = 0.522; Fig 4B). These findings show that the different gene alterations (either copy number alterations or mutations) in SMAD4 and CDKN2A drive pancreatic cells towards the malignant phenotype by perturbating the signalling pathways in which either SMAD4 or CDKN2A participate (Fig 4C and 4D).

**Fig 5:**
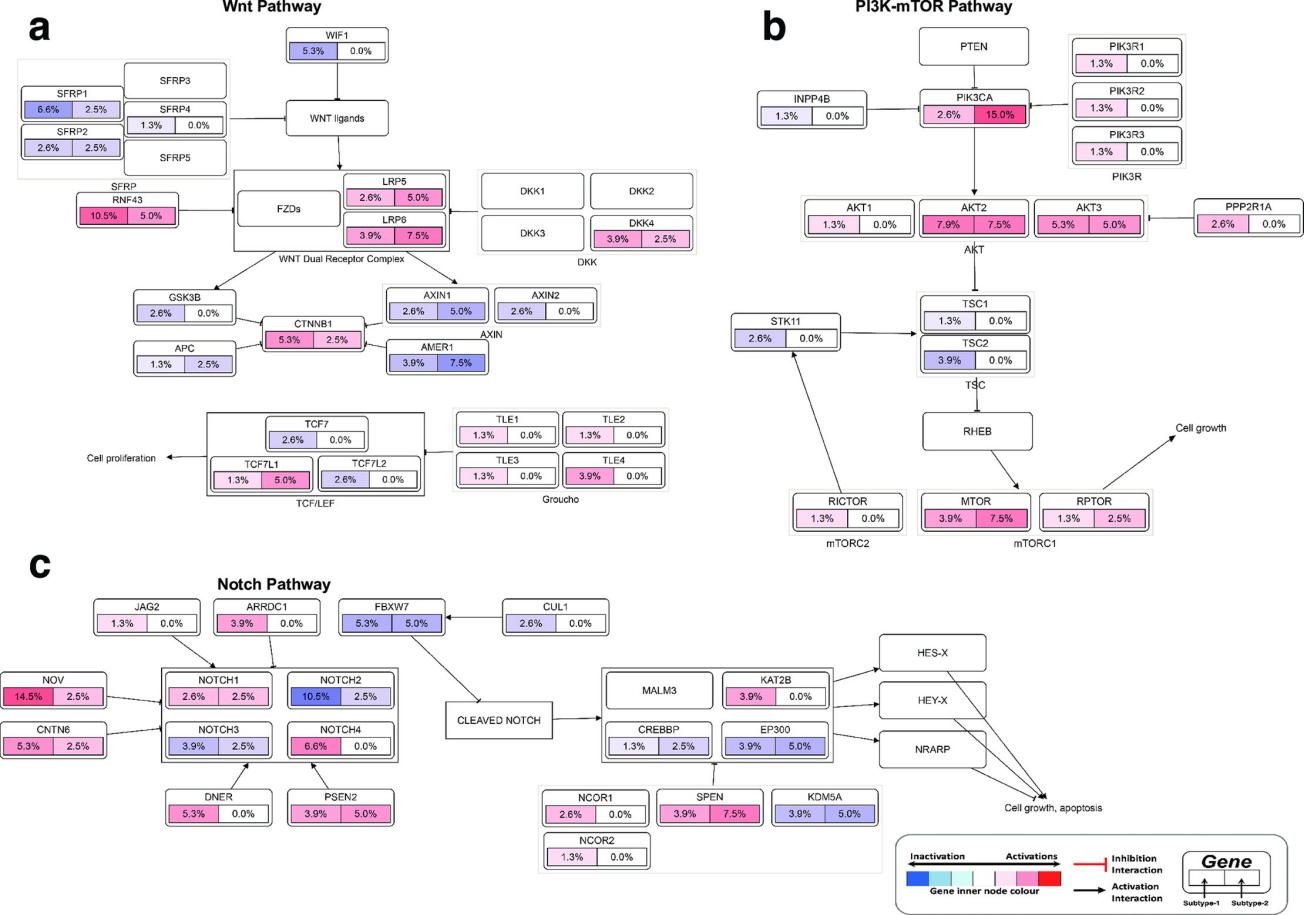
Alterations in **(a)** Wnt pathway, **(b)** PI3K-mTOR pathway and **(c)** Notch pathway. The node represents the percentage of each gene mutation and copy number alterations of (left half) subtype-1 and (right half) of subtype-2 pancreatic tumours. The nodes are coloured according to the types of genes: Blue nodes for tumour suppressor genes and red for oncogenes. The interaction types are as given in the figure legend.

We found that in pancreatic tumours, genomic alterations are common within genes that are members of various well-known cell signalling pathways. Among the pathways with gene alterations were the Receptor tyrosine kinase–Ras pathway (altered in 75% of all tumours; S1 Fig), Wnt pathway (altered in 29% of all tumours; Fig 5A), the PI3K-mTOR pathway (23%; Fig 5B) and Transforming Growth Factor Beta Signalling Pathway (48%; Fig 5C). These cell signalling pathways have been reported altered in various cancers, including those of the lungs, skin, and breast, where they have also been shown to promote oncogenesis [22,43–45]. Accordingly, we suggest that these signalling pathways may play essential roles in pancreatic cancer and may present inflexion points for targeted therapies to cure pancreatic cancer.

## Discussion

We conducted an integrated analysis of proteomics, clinical outcomes, mutations and copy number alterations of pancreatic cancer. Using machine learning, we showed that pancreatic tumours could be classified into two distinct subtypes. Patients afflicted with these disease subtypes show relatively similar demographics, suggesting that the onset of these cancer subtypes are not associated with the clinical parameters, such as age, gender and diabetes. Furthermore, we found that the disease outcomes are similar between the disease subtypes.

Among the subtype-1 tumours, we found significant enrichment for the mTOR signalling pathway (Fig 3C). Recent studies show that particular subtypes of pancreatic tumours respond well to anticancer agents that target the mTOR signalling pathway [46,47]. Therefore, we expect that the subtype-1 are possibly more responsive to drugs that target the mTOR than will the subtype-2 tumours.

Our results revealed that the mutations and copy number alteration were similar among the proteomic subtypes of pancreatic cancer. This finding may indicate that the primary genomic drivers of pancreatic oncogenes are similar among disease subtypes. The observed widespread alterations in the KRAS oncogene and TP53 tumour suppressor genes further confirm this (Fig 4A). As others have suggested, mutations in these genes likely perturb signalling through both the MAPK pathway and p53 and cell cycle pathways [48,49]. Other gene alterations, such as those we found in the CDKN2A and SMAD4 genes (Fig 4B), which we have shown to be mutually exclusive, should exert selective pressure that transforms the normal and pre-malignant cells [50–52]. Many other gene alterations in distinctive genes that participate in the same signalling pathway, such as the Notch pathway, PI3K-mTOR pathway (S1 Fig) and metastasis pathway, likely come in later and contribute toward the progression of the disease [50,53]. Recently, oncogenesis theory has been extended beyond the accumulation of genetic mutations [54,55] to include the disruption of epigenetic regulatory mechanisms and variations in miRNA expression [55–60]. Unlike gene mutations, we currently lack adequate tools to identify the driver alterations in epigenome and miRNAs [57,58,61–63]. Accordingly, we suggest that differences in the proteome (and probably the methylome, miRNA and transcriptomes) are the likely drivers of variance between the pancreatic cancer subtypes defined here and previously [10,16,17,23].

Altogether, we have revealed the clinical and molecular characteristics of two distinct subtypes of pancreatic cancer. We have further shown that one subtype exhibits hyperactivation of various pathways, including mTOR signalling, for which proteins of these pathways present a variety of potential disease subtype-specific biomarkers and drug targets.

## Methods

We obtained the TCGA project [64] datasets of 186 pancreatic cancer patients obtained from cBioPortal (http://www.cbioportal.org) [65]. We only returned and analysed 124 pancreatic cancer patients’ samples profiled using reverse-phase protein array-based (RPPA) proteomics data. Furthermore, we also utilised DNA copy number alterations and mutation data and comprehensively de-identified clinical and sample information.

### Proteomics classification of pancreatic cancer

We applied unsupervised machine learning methods to classify the pancreatic tumour samples based on the protein expression levels measured using RPPA. To evaluate the optimal number of clusters, we used the Calinski-Harabasz clustering evaluation criterion, which showed that the optimal number of clusters is two (S2A Fig) [66]. Then we applied unsupervised K-means over 1000 iterations with the squared Euclidean distance metric and chose the clustering solution with the highest average Silhouette score to define the proteomic data to identify disease subtypes [67]. Next, we visualised the clustering of the tumours; we reduced the dimensions of the proteomics measured data using Principal Component Analysis [68,69]. Finally, we plotted the first two dimensions of the principal components with points coloured based on the K-means clustering group assignment (Fig 1A). To evaluate the coherence of our K-mean clustering solution, we applied a supervised Kernel Naïve Bayes algorithm and evaluated the model’s performance using 10-fold cross-validation. Here, we used Bayesian optimisation to select the optimal machine learning hyperparameters for the Naïve Bayes algorithm (S2B Fig).

### Functional enrichment analyses

We downloaded the 2019 KEGG database and 2021 GO molecular enrichment. Then for each gene set within each database, we modified the gene sets by returning only the genes present in our proteomic dataset, thus limiting the gene background to genes only present in the proteomic data. Finally, we used Gene set enrichment analysis (GSEA) [37] to determine the KEGG pathway and GO molecular functions that are enriched for in subtype-1 tumours compared to subtype-2 tumours (see S2 File).

Next, we retrieved known protein-protein interactions from the University of California Santa Cruz Super pathway, the Kinase Enrichment Analysis, and Chromatin Immunoprecipitation Enrichment Analysis [70–72]. Then, we used these interactions to connect proteins that are members of the GO molecular function terms “Purine Ribonucleoside Triphosphate Binding” and the KEGG term mTOR signalling pathway. Then, we used yEd to visualise the overall connectivity of two resulting networks (Fig 3C and 3D).

### Analysis and mutations and copy number alterations

We evaluated the scope of gene alterations in the pancreatic subtypes using the mutation (single nucleotide polymorphisms and indels) data and copy number alterations data. First, we combined these two gene alteration data. Then we returned only genes that are associated with human cancers using information from the Sanger Consensus Cancer Gene Database [41]. Furthermore, the oncogenes and tumour suppressor genes in the gene alteration datasets were annotated using information from the UniProt Knowledgebase, the TSGene database, and the ONGene database [41,73–75]. Finally, we compared gene alterations between the disease subtypes using the Chi-square test. Also, we plotted the spectrum of genomic alterations for the fourteen most altered genes in the samples using a custom function (Fig 4A). To assess which signalling pathways, we used the PathwayMapper software [76].

### Survival analysis

The Kaplan-Meier method was used to estimate overall survival and the duration of progression-free survival between the current subtype-1 and the subtype-2 of pancreatic cancer [36]. Furthermore, the Kaplan-Meier method was applied to assess the difference in the overall survival of patients afflicted with previously described pancreatic cancer subtypes taken from the supplementary files of the publication by The Cancer Genome Atlas Network [13].

### Statistical analyses

We used MATLAB version 2020a to perform all the analyses presented here. Fisher’s exact test was used to test for associations between categorical variables. The Welch test and the Wilcoxon rank-sum tests were used to compare differences in the tumour subtypes for the continuous variables among the various categories. We considered comparison as statistically significant when p-values are < 0.05 for single comparisons and when the Benjamini-Hochberg adjusted p-values are < 0.05 for multiple comparisons.

## Supporting information

S1 Fig Alterations in Receptor tyrosine kinase–Rat Sarcoma (Ras) pathway. The node represents the percentage of each gene mutations and copy number alterations in (left half) subtype-1 and (right half) in subtype-2 pancreatic tumours. The nodes are coloured according to the types of genes: Blue nodes for tumour suppressor genes and red for oncogenes. The interaction types are as given in the figure legend.
(TIF)

S2 Fig **(a)** Evaluating the optimum number of clusters: The plot displays the Calinski-Harabasz evaluation method [66]. The optimum number of clusters is the number of cluster values that correspond to the highest Calinski-Harabasz value. In this case, the optimum number of clusters is two. **(b)** Range of values assessed by the Bayesian optimisation objective function to select the optimal machine learning hyperparameters for the Kernel naïve Bayes supervised learning model [77,78].
(TIF)

S1 File Differential expression results for proteins between subtype-1 and subtype-2 tumours of pancreatic cancer. (XLSX)

S2 File KEGG pathways and Gene Ontology Molecular Function terms that are significantly enriched for in each subtype-1 tumours compared to subtype-2 tumours of pancreatic cancer. (XLSX)

S3 File Gene alteration spectrum between the subtype-1 and subtype-2 tumours of pancreatic cancer. (XLSX)

S4 File (DOCX)

S5 File (DOCX)

## Abbreviations

TCGA: The Cancer Genome Atlas
RPPA: Reverse Phase Protein Array
GO: Gene Ontology
P.C.A.: Principal Component Analysis

## References

[1] Chang DK, Grimmond SM, Biankin A V. Pancreatic cancer genomics. Curr Opin Genet Dev 2014;24:74–81. doi: 10.1016/j.gde.2013.12.001 24480245

[2] Waddell N, Pajic M, Patch A-M, Chang DK, Kassahn KS, Bailey P, et al. Whole genomes redefine the mutational landscape of pancreatic cancer. Nature 2015;518:495–501. doi: 10.1038/nature14169 25719666 PMC4523082

[3] Bouvier AM, David M, Jooste V, Chauvenet M, Lepage C, Faivre J. Rising incidence of pancreatic cancer in France. Pancreas 2010;39:1243–6. doi: 10.1097/MPA.0b013e3181e1d5b3 20881902

[4] Wong MCS, Jiang JY, Liang M, Fang Y, Yeung MS, Sung JJY. Global temporal patterns of pancreatic cancer and association with socioeconomic development. Sci Rep 2017;7:1–9. doi: 10.1038/s41598-016-0028-x 28600530 PMC5466634

[5] Belpomme D, Irigaray P, Sasco AJ, Newby JA, Howard V, Clapp R, et al. The growing incidence of cancer: Role of lifestyle and screening detection (Review). Int J Oncol 2007;30:1037–49. doi: 10.3892/ijo.30.5.1037 17390005

[6] Ellison EC, Pawlik TM, Way DP, Satiani B, Williams TE. The impact of the aging population and incidence of cancer on future projections of general surgical workforce needs. Surg (United States) 2018;163:553–9. doi: 10.1016/j.surg.2017.09.035 29179915

[7] Noll EM, Eisen C, Stenzinger A, Espinet E, Muckenhuber A, Klein C, et al. CYP3A5 mediates basal and acquired therapy resistance in different subtypes of pancreatic ductal adenocarcinoma. Nat Med 2016;22:278–87. doi: 10.1038/nm.4038 26855150 PMC4780258

[8] Puleo F, Nicolle R, Blum Y, Cros J, Marisa L, Demetter P, et al. Stratification of Pancreatic Ductal Adenocarcinomas Based on Tumor and Microenvironment Features. Gastroenterology 2018;155:1999–2013.e3. doi: 10.1053/j.gastro.2018.08.033 30165049

[9] Moffitt RA, Marayati R, Flate EL, Volmar KE, Loeza SGH, Hoadley KA, et al. Virtual microdissection identifies distinct tumor- and stroma-specific subtypes of pancreatic ductal adenocarcinoma. Nat Genet 2015;47:1168–78. doi: 10.1038/ng.3398 26343385 PMC4912058

[10] Lomberk G, Blum Y, Nicolle R, Nair A, Gaonkar KS, Marisa L, et al. Distinct epigenetic landscapes underlie the pathobiology of pancreatic cancer subtypes. Nat Commun 2018;9:1–10. doi: 10.1038/s41467-017-02088-w 29773832 PMC5958058

[11] Boikos SA, Pappo AS, Killian JK, LaQuaglia MP, Weldon CB, George S, et al. Molecular subtypes of KIT/PDGFRA wild-type gastrointestinal stromal tumors a report from the national institutes of health gastrointestinal stromal tumor clinic. JAMA Oncol 2016;2:922–8. doi: 10.1001/jamaoncol.2016.0256 27011036 PMC5472100

[12] Ogino S, Fuchs CS, Giovannucci E. How many molecular subtypes? Implications of the unique tumor principle in personalized medicine. Expert Rev Mol Diagn 2012;12:621–8. doi: 10.1586/erm.12.46 22845482 PMC3492839

[13] Cancer Genome Atlas Research Network. Electronic address: andrew_aguirre@dfci.harvard.edu TCGAR, Cancer Genome Atlas Research Network. Integrated Genomic Characterization of Pancreatic Ductal Adenocarcinoma. Cancer Cell 2017;32:185-203.e13. doi: 10.1016/j.ccell.2017.07.007 PMC5964983 28810144

[14] Sinkala M, Mulder N, Martin DP. Integrative landscape of dysregulated signaling pathways of clinically distinct pancreatic cancer subtypes. Oncotarget 2018;9. doi: 10.18632/oncotarget.25632 30018740 PMC6044387

[15] Dzobo K, Sinkala M. Cancer Stem Cell Marker CD44 Plays Multiple Key Roles in Human Cancers: Immune Suppression/Evasion, Drug Resistance, Epithelial–Mesenchymal Transition, and Metastasis. https://HomeLiebertpubCom/Omi 2021;25:313–32. doi: 10.1089/omi.2021.0025 33961518

[16] Bailey P, Chang DK, Nones K, Johns AL, Patch A-M, Gingras M-C, et al. Genomic analyses identify molecular subtypes of pancreatic cancer. Nature 2016;531:47–52. doi: 10.1038/nature16965 26909576

[17] Collisson EA, Sadanandam A, Olson P, Gibb WJ, Truitt M, Gu S, et al. Subtypes of pancreatic ductal adenocarcinoma and their differing responses to therapy. Nat Med 2011;17:500–3. doi: 10.1038/nm.2344 21460848 PMC3755490

[18] Sinkala M, Mulder N, Martin D. Machine Learning and Network Analyses Reveal Disease Subtypes of Pancreatic Cancer and their Molecular Characteristics. Sci Rep 2020;10. doi: 10.1038/s41598-020-58290-2 31988390 PMC6985164

[19] Kafita D, Daka V, Nkhoma P, Zulu M, Zulu E, Tembo R, et al. High ELF4 expression in human cancers is associated with worse disease outcomes and increased resistance to anticancer drugs. PLoS One 2021;16:e0248984. doi: 10.1371/journal.pone.0248984 33836003 PMC8034723

[20] Sinkala M, Zulu M, Nkhoma P, Kafita D, Zulu E, Tembo R, et al. A Systems Approach Identifies Key Regulators of HPV-Positive Cervical Cancer Citation. Syst Approach Identifies Key Regul HPV-Positive Cerv Cancer J Bioinforma Syst Biol 2021;4:33–49. doi: 10.26502/jbsb.5107020

[21] Hao K, Tian XD, Qin CF, Xie XH, Yang YM. Hedgehog signaling pathway regulates human pancreatic cancer cell proliferation and metastasis. Oncol Rep 2013;29:1124–32. doi: 10.3892/or.2012.2210 23292285

[22] Yabuuchi S, Pai SG, Campbell NR, De Wilde RF, De Oliveira E, Korangath P, et al. Notch signaling pathway targeted therapy suppresses tumor progression and metastatic spread in pancreatic cancer. Cancer Lett 2013;335:41–51. doi: 10.1016/j.canlet.2013.01.054 23402814 PMC3665739

[23] Raphael BJ, Hruban RH, Aguirre AJ, Moffitt RA, Yeh JJ, Stewart C, et al. Integrated Genomic Characterization of Pancreatic Ductal Adenocarcinoma. Cancer Cell 2017;32:185–203.e13. doi: 10.1016/j.ccell.2017.07.007 28810144 PMC5964983

[24] Sidaway P. Pancreatic cancer: TCGA data reveal a highly heterogeneous disease. Nat Rev Clin Oncol 2017;14:648. doi: 10.1038/nrclinonc.2017.146 28857073

[25] Zhang H, Liu T, Zhang Z, Payne SH, Zhang B, McDermott JE, et al. Integrated Proteogenomic Characterization of Human High-Grade Serous Ovarian Cancer. Cell 2016;166:755–65. doi: 10.1016/j.cell.2016.05.069 27372738 PMC4967013

[26] Mertins P, Mani DR, Ruggles K V., Gillette MA, Clauser KR, Wang P, et al. Proteogenomics connects somatic mutations to signalling in breast cancer. Nature 2016;534:55–62. doi: 10.1038/nature18003 27251275 PMC5102256

[27] Sinkala M, Mulder N, Patrick Martin D. Metabolic gene alterations impact the clinical aggressiveness and drug responses of 32 human cancers. Commun Biol 2019;2. doi: 10.1038/s42003-019-0666-1 31754644 PMC6856368

[28] Zhang S, Chang W, Wu H, Wang Y, Gong Y, Zhao Y, et al. Pan‐cancer analysis of iron metabolic landscape across the Cancer Genome Atlas. J Cell Physiol 2020;235:1013–24. doi: 10.1002/jcp.29017 31240715

[29] Chen F, Zhang Y, Varambally S, Creighton CJ. Molecular correlates of metastasis by systematic pan-cancer analysis across the cancer genome atlas. Mol Cancer Res 2019;17:476–87. doi: 10.1158/1541-7786.MCR-18-0601 30401717 PMC6359982

[30] Kim J, Kwiatkowski D, McConkey DJ, Meeks JJ, Freeman SS, Bellmunt J, et al. The Cancer Genome Atlas Expression Subtypes Stratify Response to Checkpoint Inhibition in Advanced Urothelial Cancer and Identify a Subset of Patients with High Survival Probability. Eur Urol 2019;75:961–4. doi: 10.1016/j.eururo.2019.02.017 30851984

[31] Pan S, Brentnall TA, Chen R. Proteome alterations in pancreatic ductal adenocarcinoma. Cancer Lett 2020;469:429–36. doi: 10.1016/j.canlet.2019.11.020 31734355 PMC9017243

[32] Ansari D, Torén W, Zhou Q, Hu D, Andersson R. Proteomic and genomic profiling of pancreatic cancer. Cell Biol Toxicol 2019;35:333–43. doi: 10.1007/s10565-019-09465-9 30771135 PMC6757097

[33] Sinkala M, Nkhoma P, Mulder N, Martin DP. Integrated molecular characterisation of the MAPK pathways in human cancers reveals pharmacologically vulnerable mutations and gene dependencies. Commun Biol 2021;4:1–16. doi: 10.1038/s42003-020-01566-0 33398072 PMC7782843

[34] ACM Special Interest Group for Algorithms and Computation Theory. D, SIAM Activity Group on Discrete Mathematics. S, Association for Computing Machinery., Society for Industrial and Applied Mathematics. Proceedings of the eighteenth annual ACM-SIAM Symposium on Discrete Algorithms. Association for Computing Machinery; 2007.

[35] Hastie Trevor, Tibshirani Robert, Friedman J. The Elements of Statistical Learning The Elements of Statistical LearningData Mining, Inference, and Prediction, Second Edition. 2009. doi: 10.1007/978-0-387-84858-7

[36] Goel MK, Khanna P, Kishore J. Understanding survival analysis: Kaplan-Meier estimate. Int J Ayurveda Res 2010;1:274–8. doi: 10.4103/0974-7788.76794 21455458 PMC3059453

[37] Subramanian A, Tamayo P, Mootha VK, Mukherjee S, Ebert BL, Gillette MA, et al. Gene set enrichment analysis: a knowledge-based approach for interpreting genome-wide expression profiles. Proc Natl Acad Sci U S A 2005;102:15545–50. doi: 10.1073/pnas.0506580102 16199517 PMC1239896

[38] Ishimura N, Yamasawa K, Rumi MAK, Kadowaki Y, Ishihara S, Amano Y, et al. BRAF and K-ras gene mutations in human pancreatic cancers. Cancer Lett 2003;199:169–73. doi: 10.1016/s0304-3835(03)00384-7 12969789

[39] Heidorn SJ, Milagre C, Whittaker S, Nourry A, Niculescu-Duvas I, Dhomen N, et al. Kinase-Dead BRAF and Oncogenic RAS Cooperate to Drive Tumor Progression through CRAF. Cell 2010;140:209–21. doi: 10.1016/j.cell.2009.12.040 20141835 PMC2872605

[40] Testa JR, Bellacosa A. AKT plays a central role in tumorigenesis. Proc Natl Acad Sci U S A 2001;98:10983–5. doi: 10.1073/pnas.211430998 11572954 PMC58668

[41] Forbes SA, Beare D, Gunasekaran P, Leung K, Bindal N, Boutselakis H, et al. COSMIC: exploring the world’s knowledge of somatic mutations in human cancer. Nucleic Acids Res 2015;43:D805–11. doi: 10.1093/nar/gku1075 25355519 PMC4383913

[42] Witkiewicz AK, McMillan EA, Balaji U, Baek G, Lin W-C, Mansour J, et al. Whole-exome sequencing of pancreatic cancer defines genetic diversity and therapeutic targets. Nat Commun 2015;6:6744. doi: 10.1038/ncomms7744 25855536 PMC4403382

[43] McCubrey JA, Steelman LS, Bertrand FE, Davis NM, Sokolosky M, Abrams SL, et al. GSK-3 as potential target for therapeutic intervention in cancer. Oncotarget 2014;5:2881–911. doi: 10.18632/oncotarget.2037 24931005 PMC4102778

[44] Zhang YE. Non-Smad pathways in TGF-beta signaling. Cell Res 2009;19:128–39. doi: 10.1038/cr.2008.328 19114990 PMC2635127

[45] Balmanno K, Cook SJ. Tumour cell survival signalling by the ERK1/2 pathway. Cell Death Differ 2009;16:368–77. doi: 10.1038/cdd.2008.148 18846109

[46] Larbouret C, Gaborit N, Chardès T, Coelho M, Campigna E, Bascoul-Mollevi C, et al. In pancreatic carcinoma, dual EGFR/HER2 targeting with cetuximab/trastuzumab is more effective than treatment with trastuzumab/erlotinib or lapatinib alone: Implication of receptors’ down-regulation and dimers’ disruption. Neoplasia 2012;14:121–30. doi: 10.1593/neo.111602 22431920 PMC3306257

[47] Singla S, Pippin JA, Drebin JA. Dual ErbB1 and ErbB2 receptor tyrosine kinase inhibition exerts synergistic effect with conventional chemotherapy in pancreatic cancer. Oncol. Rep., vol. 28, 2012, p. 2211–6. doi: 10.3892/or.2012.2053 23007710

[48] MacGregor-Das AM, Iacobuzio-Donahue CA. Molecular pathways in pancreatic carcinogenesis. J Surg Oncol 2013;107:8–14. doi: 10.1002/jso.23213 22806689 PMC3661191

[49] Mccleary-Wheeler AL, Mcwilliams R, Fernandez-Zapico ME. Aberrant signaling pathways in pancreatic cancer: A two compartment view. Mol Carcinog 2012;51:25–39. doi: 10.1002/mc.20827 22162229 PMC3253704

[50] Notta F, Chan-Seng-Yue M, Lemire M, Li Y, Wilson GW, Connor AA, et al. A renewed model of pancreatic cancer evolution based on genomic rearrangement patterns. Nature 2016;538:378–82. doi: 10.1038/nature19823 27732578 PMC5446075

[51] Makohon-Moore A, Iacobuzio-Donahue CA. Pancreatic cancer biology and genetics from an evolutionary perspective. Nat Rev Cancer 2016;16:553–65. doi: 10.1038/nrc.2016.66 27444064 PMC5739515

[52] Yachida S, Jones S, Bozic I, Antal T, Leary R, Fu B, et al. Distant metastasis occurs late during the genetic evolution of pancreatic cancer. Nature 2010;467:1114–7. doi: 10.1038/nature09515 20981102 PMC3148940

[53] Yachida S, Iacobuzio-Donahue CA. Evolution and dynamics of pancreatic cancer progression. Oncogene 2013;32:5253–60. doi: 10.1038/onc.2013.29 23416985 PMC3823715

[54] Hanahan D, Weinberg RA. Hallmarks of cancer: The next generation. vol. 144. Elsevier; 2011. doi: 10.1016/j.cell.2011.02.013 21376230

[55] Duesberg P, Rausch C, Rasnick D, Hehlmann R, Woodgate R, Goodman MF, et al. Genetic instability of cancer cells is proportional to their degree of aneuploidy. Proc Natl Acad Sci 1998;95:13692–7. doi: 10.1073/pnas.95.23.13692 9811862 PMC24881

[56] Coyle KM, Boudreau JE, Marcato P. Genetic Mutations and Epigenetic Modifications: Driving Cancer and Informing Precision Medicine. Biomed Res Int 2017;2017:9620870. doi: 10.1155/2017/9620870 28685150 PMC5480027

[57] Sharma S, Kelly TK, Jones PA. Epigenetics in cancer. Carcinogenesis 2010;31:27–36. doi: 10.1093/carcin/bgp220 19752007 PMC2802667

[58] Reddy KB. MicroRNA (miRNA) in cancer. Cancer Cell Int 2015;15:38. doi: 10.1186/s12935-015-0185-1 25960691 PMC4424445

[59] Mishra NK, Guda C. Genome-wide DNA methylation analysis reveals molecular subtypes of pancreatic cancer. Oncotarget 2017;8:28990–9012. doi: 10.18632/oncotarget.15993 28423671 PMC5438707

[60] Khatri I, Ganguly K, Sharma S, Carmicheal J, Kaur S, Batra SK, et al. Systems Biology Approach to Identify Novel Genomic Determinants for Pancreatic Cancer Pathogenesis. Sci Rep 2019;9:123. doi: 10.1038/s41598-018-36328-w 30644396 PMC6333820

[61] Kazanets A, Shorstova T, Hilmi K, Marques M, Witcher M. Epigenetic silencing of tumor suppressor genes: Paradigms, puzzles, and potential. Biochim Biophys Acta—Rev Cancer 2016;1865:275–88. doi: 10.1016/J.BBCAN.2016.04.001 27085853

[62] Chatterjee A, Rodger EJ, Eccles MR. Epigenetic drivers of tumourigenesis and cancer metastasis. Semin Cancer Biol 2018;51:149–59. doi: 10.1016/j.semcancer.2017.08.004 28807546

[63] Shen H, Laird PW. Interplay between the Cancer Genome and Epigenome. Cell 2013;153:38–55. doi: 10.1016/j.cell.2013.03.008 23540689 PMC3648790

[64] Chang K, Creighton CJ, Davis C, Donehower L, Drummond J, Wheeler D, et al. The Cancer Genome Atlas Pan-Cancer analysis project. Nat Genet 2013;45:1113–20. doi: 10.1038/ng.2764 24071849 PMC3919969

[65] Cerami E, Gao J, Dogrusoz U, Gross BE, Sumer SO, Aksoy BA, et al. The cBio cancer genomics portal: an open platform for exploring multidimensional cancer genomics data. Cancer Discov 2012;2:401–4. doi: 10.1158/2159-8290.CD-12-0095 22588877 PMC3956037

[66] Caliñski T, Harabasz J. A Dendrite Method Foe Cluster Analysis. Commun Stat 1974;3:1–27. doi: 10.1080/03610927408827101

[67] Wu Y, Ianakiev K, Govindaraju V. Improved k-nearest neighbor classification. Pattern Recognit 2002;35:2311–8. doi: 10.1016/S0031-3203(01)00132-7

[68] Abdi H, Williams LJ. Principal component analysis. Wiley Interdiscip Rev Comput Stat 2010;2:433–59. doi: 10.1002/wics.101

[69] Jolliffe I. Principal Component Analysis. Int. Encycl. Stat. Sci., Berlin, Heidelberg: Springer Berlin Heidelberg; 2011, p. 1094–6. doi: 10.1007/978-3-642-04898-2_455

[70] Lachmann A, Ma’ayan A. KEA: kinase enrichment analysis. Bioinformatics 2009;25:684–6. doi: 10.1093/bioinformatics/btp026 19176546 PMC2647829

[71] Lachmann A, Xu H, Krishnan J, Berger SI, Mazloom AR, Ma’ayan A. ChEA: transcription factor regulation inferred from integrating genome-wide ChIP-X experiments. Bioinformatics 2010;26:2438–44. doi: 10.1093/bioinformatics/btq466 20709693 PMC2944209

[72] Wong CK, Vaske CJ, Ng S, Sanborn JZ, Benz SC, Haussler D, et al. The UCSC Interaction Browser: multidimensional data views in pathway context. Nucleic Acids Res 2013;41:W218–24. doi: 10.1093/nar/gkt473 23748957 PMC3692096

[73] UniProt: the universal protein knowledgebase. Nucleic Acids Res 2017;45:D158–69. doi: 10.1093/nar/gkw1099 27899622 PMC5210571

[74] Zhao M, Kim P, Mitra R, Zhao J, Zhao Z. TSGene 2.0: an updated literature-based knowledgebase for tumor suppressor genes. Nucleic Acids Res 2016;44:D1023–31. doi: 10.1093/nar/gkv1268 26590405 PMC4702895

[75] Liu Y, Sun J, Zhao M. ONGene: A literature-based database for human oncogenes. J Genet Genomics 2017;44:119–21. doi: 10.1016/j.jgg.2016.12.004 28162959

[76] Bahceci I, Dogrusoz U, La KC, Babur Ö, Gao J, Schultz N. PathwayMapper: a collaborative visual web editor for cancer pathways and genomic data. Bioinformatics 2017;33:2238–40. doi: 10.1093/bioinformatics/btx149 28334343 PMC5859976

[77] Gelbart MA, Snoek J, Adams RP. Bayesian Optimization with Unknown Constraints 2014.

[78] Snoek J, Larochelle H, Adams RP. Practical Bayesian Optimization of Machine Learning Algorithms 2012:2951–9.

