# Supplementary figures and images for: Proteogenomic analysis of pancreatic cancer subtypes
Source: PLoS One. 2021 Sep 10;16(9):e0257084. doi: 10.1371/journal.pone.0257084 (PMC8432812; doi:10.1371/journal.pone.0257084)

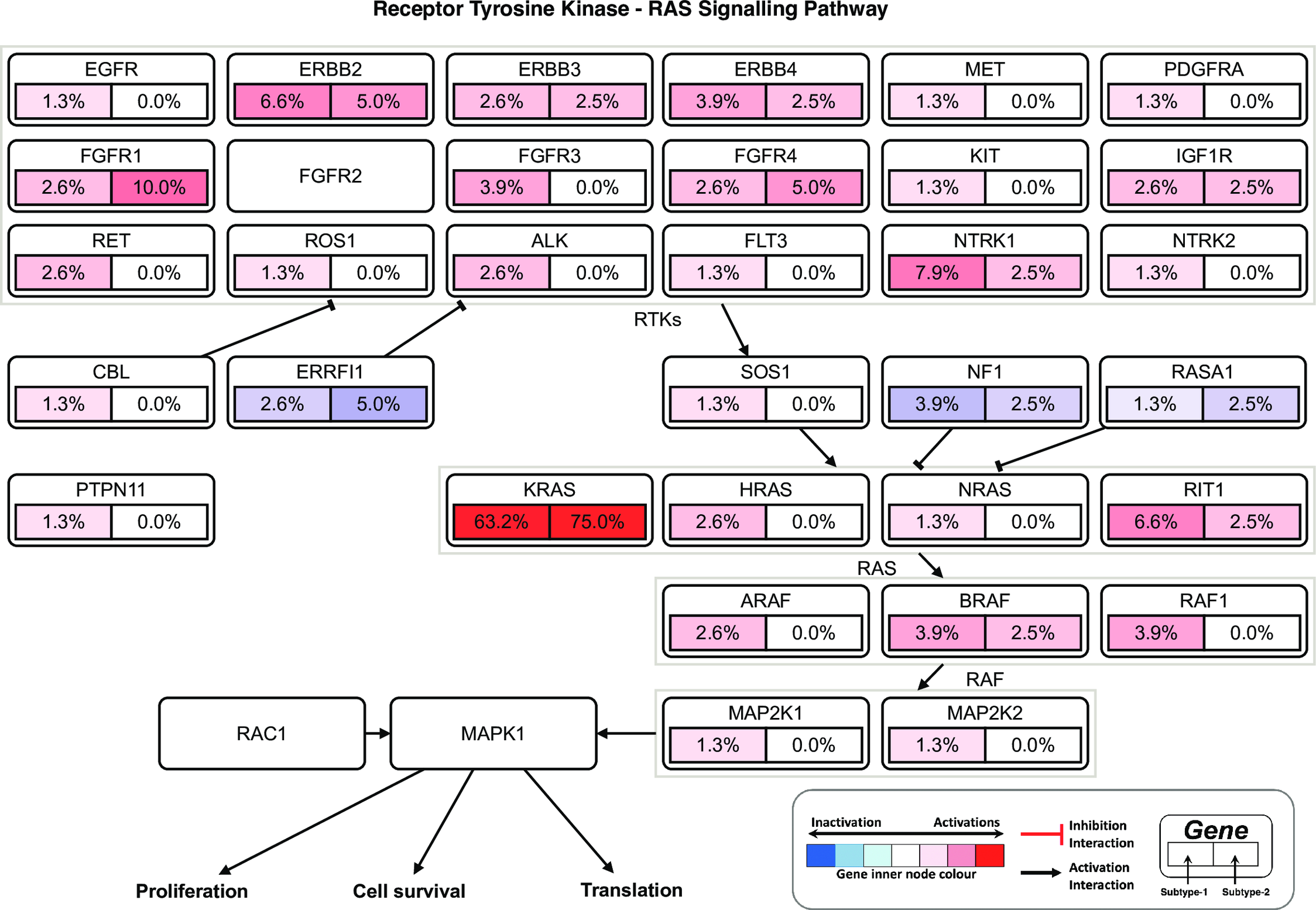

Supplement: S1 Fig — The node represents the percentage of each gene mutations and copy number alterations in (left half) subtype-1 and (right half) in subtype-2 pancreatic tumours. The nodes are coloured according to the types of genes: Blue nodes for tumour suppressor genes and red for oncogenes. The interaction types are as given in the figure legend. (TIF) [file pone.0257084.s001.tif]

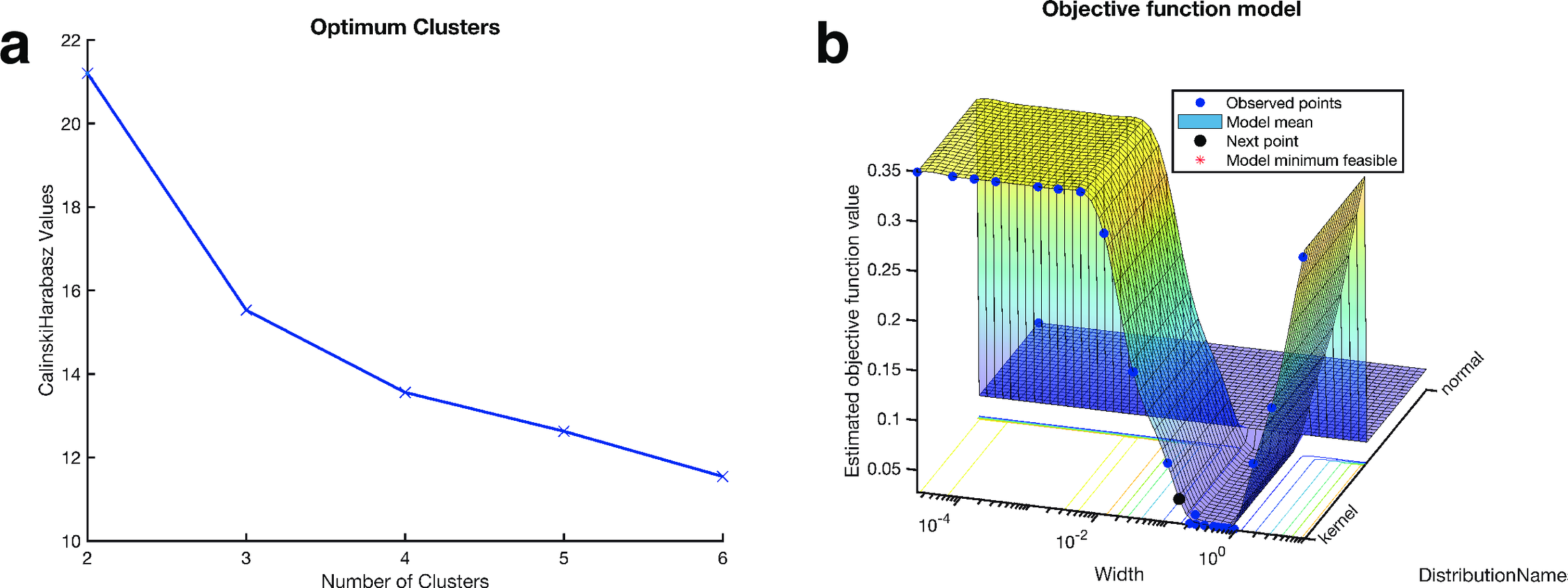

Supplement: S2 Fig — (a) Evaluating the optimum number of clusters: The plot displays the Calinski-Harabasz evaluation method [66]. The optimum number of clusters is the number of cluster values that correspond to the highest Calinski-Harabasz value. In this case, the optimum number of clusters is two. (b) Range of values assessed by the Bayesian optimisation objective function to select the optimal machine learning hyperparameters for the Kernel naïve Bayes supervised learning model [77,78]. (TIF) [file pone.0257084.s002.tif]
