# Supplementary material for: Proteogenomic analysis of pancreatic cancer subtypes
Source: PLoS One. 2021 Sep 10;16(9):e0257084. doi: 10.1371/journal.pone.0257084 (PMC8432812; doi:10.1371/journal.pone.0257084)
Supplement: S4 File — (DOCX) [file pone.0257084.s006.docx]

# Supplemental Information titles and legends


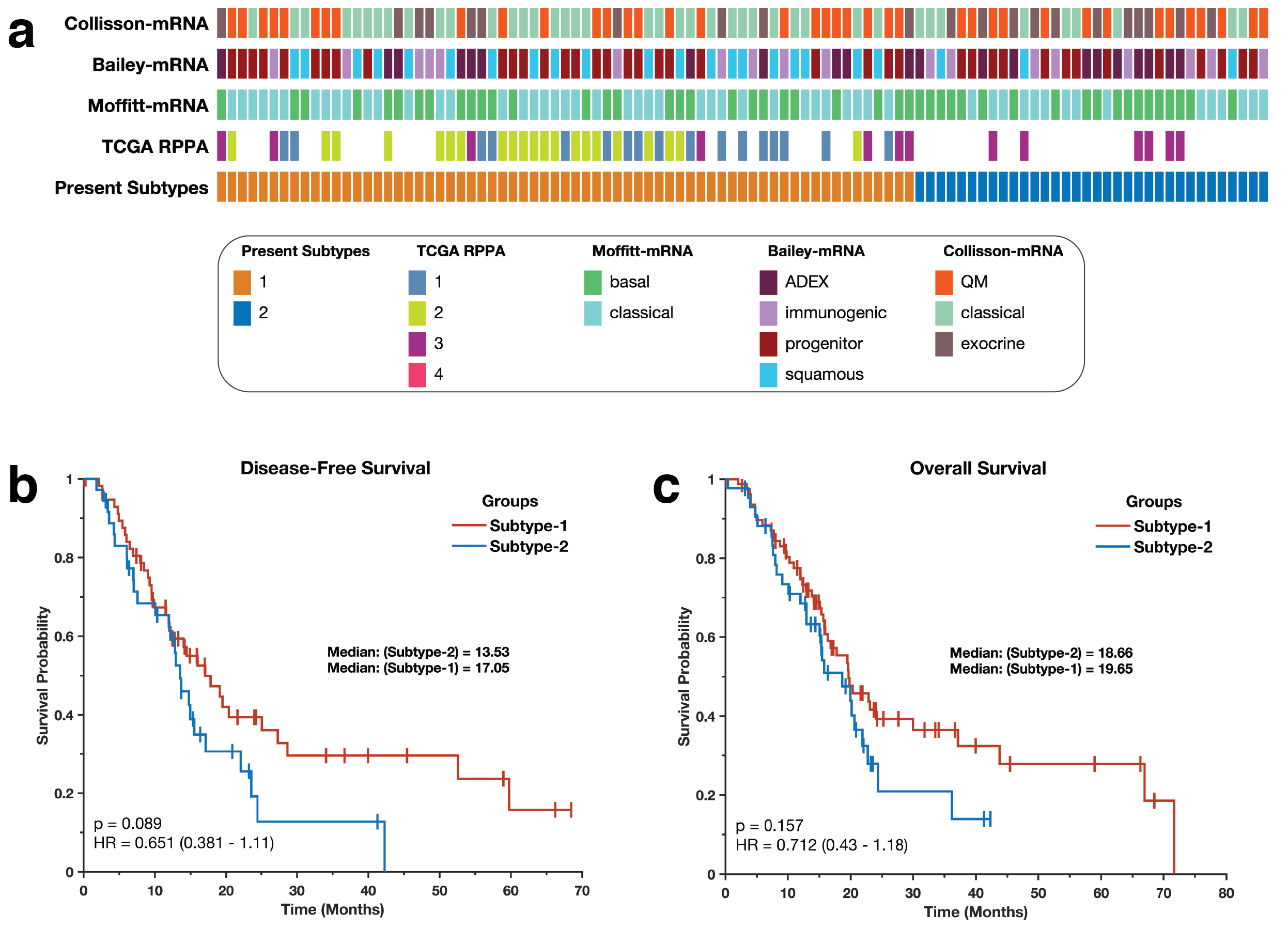


**Figure S1:** **(a)** Comparison between the current proteomic based classification of pancreatic cancers to other classification schemes from top to bottom: mRNA-based classification schemes established by Collisson et al.; Bailey et al.; and Moffitt et al., and the TCGA’s (Raphael et al., 2017) RPPA classification scheme. **(b)** Kaplan-Meier curve of the disease-free survival months of patients afflicted by each of the pancreatic cancer subtypes (**c)** Kaplan-Meier curve of the overall survival months of patients afflicted by each pancreatic cancer subtypes.


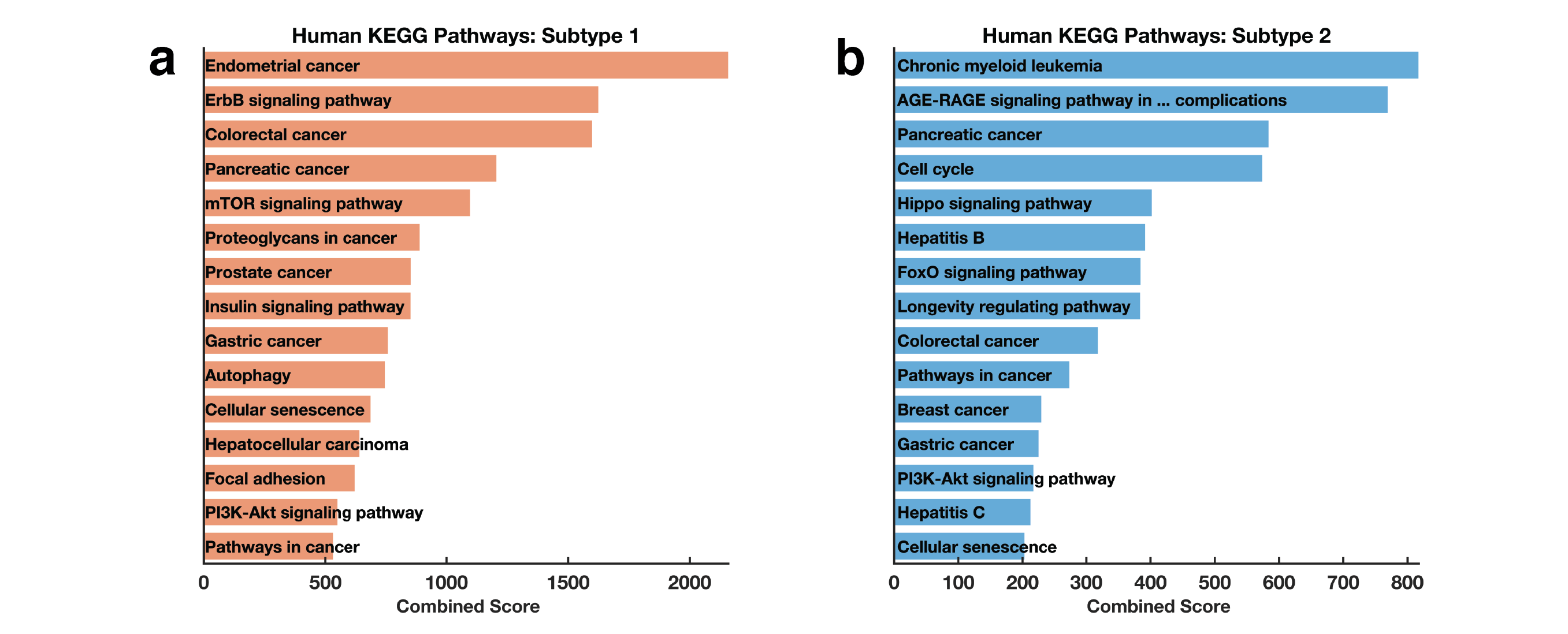


**Figure S2:** KEGG pathways: showing the top-ranked human KEGG pathway [1] enriched for **(a)** disease subtype-1 and **(b)** disease subtype-2 of pancreatic cancer based on the protein expression levels.


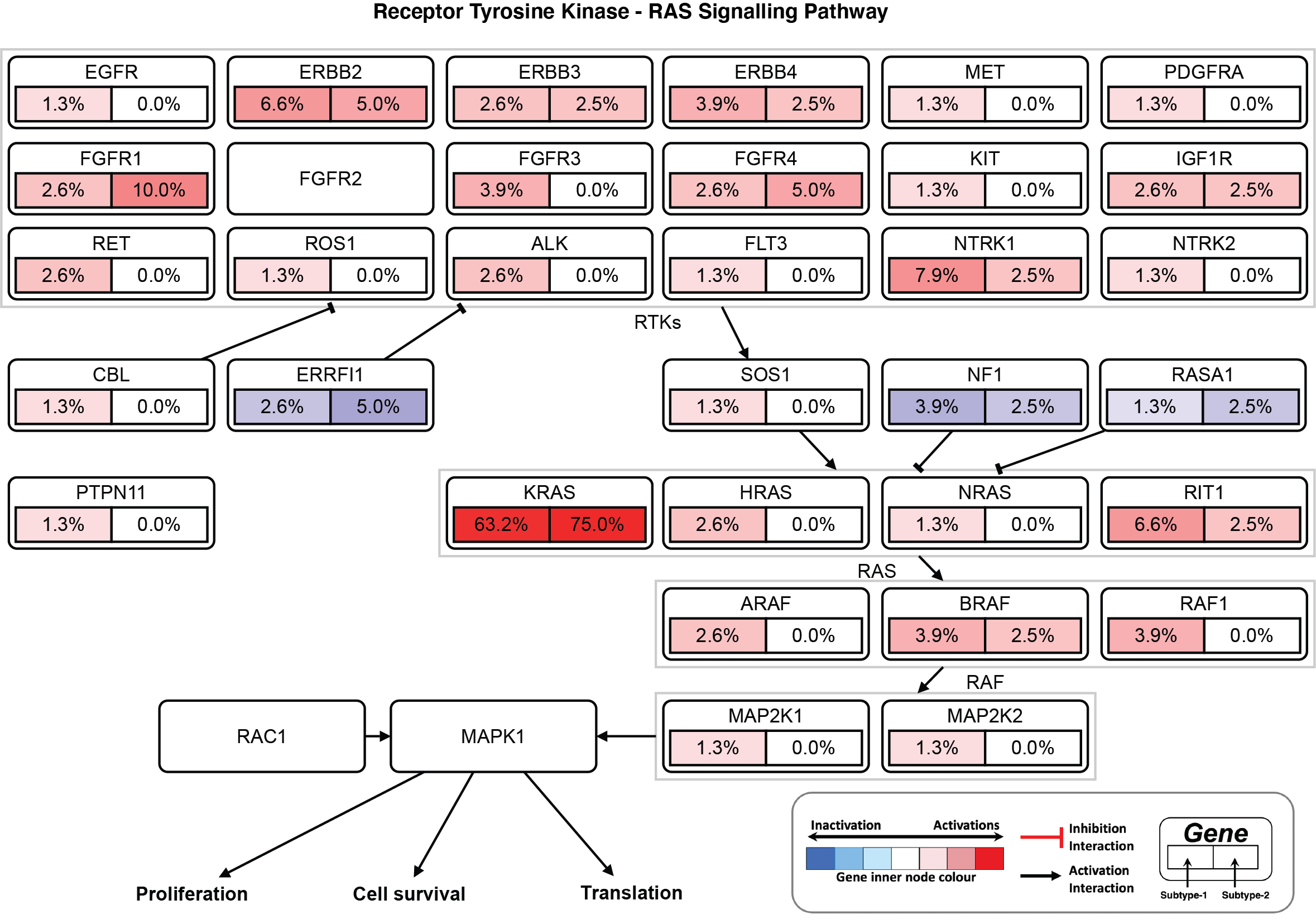


**Figure S3:** Alterations in Receptor Tyrosine Kinase – Rat Sarcoma (Ras) pathway. The node represents the percentage of each gene mutations and copy number alterations in (left half) subtype-1 and (right half) in subtype-2 pancreatic tumours. The nodes are coloured according to the types of genes: blue nodes for tumour suppressor genes and red for oncogenes. The interaction types are as given in the figure legend.


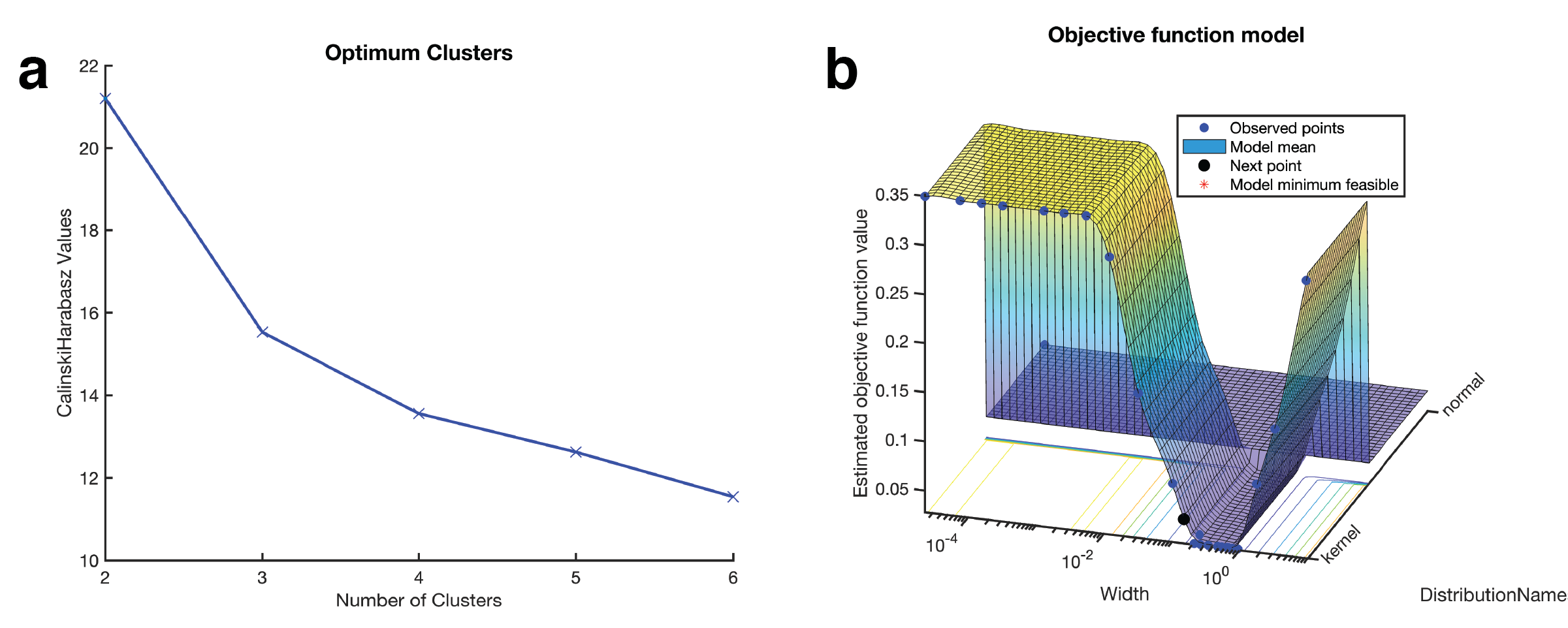


**Figure S4:** **(a)** Evaluating the optimum number of clusters: the plot displays the Calinski-Harabasz evaluation method [2]. The optimum number of clusters is the number of cluster values that correspond to the highest Calinski-Harabasz value. In this case, the optimum number of clusters is two. **(b)** Range of values assessed by the Bayesian optimisation objective function to select the optimal machine learning hyperparameters for the Kernel naïve Bayes supervised learning model [3,4].

# Supplemental Files

**Supplementary File 1:** Differential expression results for proteins between subtype-1 and subtype-2 tumours of pancreatic cancer.

**Supplementary File 2:** KEGG pathways [1] and Gene Ontology [5] Molecular Function terms that are significantly enriched for in each subtype of pancreatic cancer.

# References

[1] Kanehisa M, Sato Y, Kawashima M, Furumichi M, Tanabe M. KEGG as a reference resource for gene and protein annotation. Nucleic Acids Res 2016;44:D457–62. doi:10.1093/nar/gkv1070.

[2] Caliñski T, Harabasz J. A Dendrite Method Foe Cluster Analysis. Commun Stat 1974;3:1–27. doi:10.1080/03610927408827101.

[3] Gelbart MA, Snoek J, Adams RP. Bayesian Optimization with Unknown Constraints 2014.

[4] Snoek J, Larochelle H, Adams RP. Practical Bayesian Optimization of Machine Learning Algorithms 2012:2951–9.

[5] Gene Ontology Consortium: going forward. Nucleic Acids Res 2015;43:D1049–56. doi:10.1093/nar/gku1179.
